# A transcriptomic reporter assay employing neutrophils to measure immunogenic activity of septic patients’ plasma

**DOI:** 10.1186/1479-5876-12-65

**Published:** 2014-03-11

**Authors:** Prasong Khaenam, Darawan Rinchai, Matthew C Altman, Laurent Chiche, Surachat Buddhisa, Chidchamai Kewcharoenwong, Duangchan Suwannasaen, Michael Mason, Elizabeth Whalen, Scott Presnell, Wattanachai Susaengrat, Kimberly O’Brien, Quynh-Ahn Nguyen, Vivian Gersuk, Peter S Linsley, Ganjana Lertmemongkolchai, Damien Chaussabel

**Affiliations:** 1 Systems Immunology Division, Benaroya Research Institute, 1201 Ninth Avenue, Seattle, WA 98101, USA; 2 Centre for Research and Development of Medical Diagnostic Laboratories (CMDL), Faculty of Associated Medical Sciences, Khon Kaen University, 123 Friendship Highway, Muang, Khon Kaen 40002, Thailand; 3 Department of Internal Medicine, Hospital La Conception, 147 Blvd Baille, Marseille 13005, France; 4 Department of Internal Medicine, Khon Kaen Regional Hospital, 54 Srichan Road, Muang, Khon Kaen 40000, Thailand

**Keywords:** Transcriptomic reporter assay, Sepsis, Plasma, Microarray, Systems immunology

## Abstract

**Background:**

There are diverse molecules present in blood plasma that regulate immune functions and also present a potential source of disease biomarkers and therapeutic targets. Genome-wide profiling has become a powerful method for assessing immune responses on a systems scale, but technologies that can measure the plasma proteome still face considerable challenges. An alternative approach to direct proteome assessment is to measure transcriptome responses in reporter cells exposed *in vitro* to plasma. In this report we describe such a “transcriptomic reporter assay” to assess plasma from patients with sepsis, which is a common and severe systemic infectious process for which physicians lack efficient diagnostic or prognostic markers.

**Methods:**

Plasma samples collected from patients with culture-confirmed bacterial sepsis and uninfected healthy controls were used to stimulate three separate cell types – neutrophils, peripheral blood mononuclear cells, and monocyte-derived dendritic cells. Whole genome microarrays were generated from stimulated cells to assess transcriptional responses. Unsupervised analysis and enriched functional networks were evaluated for each cell type. Principal component analyses were used to assess variability in responses. A random K-nearest neighbor – feature selection algorithm was used to identify markers predictive of sepsis severity, which were then validated in an independent data set.

**Results:**

Neutrophils demonstrated the most distinct response to plasma from septic patients with 709 genes showing altered expression profiles, many of which are involved in established immunologic pathways. The amplitude of the neutrophil transcriptomic response was shown to be correlated with sepsis severity in two independent sets of patients comprised of 64 total septic patients. A subset of 30 transcripts selected using one set of patients was demonstrated to have a high degree of accuracy (82-90%) in predicting sepsis severity and outcomes in the other independent set. This subset included several genes previously established in sepsis pathogenesis as well as novel genes.

**Conclusions:**

These results demonstrate both the suitability and potential clinical relevance of a neutrophil reporter assay for studying plasma, in this case from septic patients. The distinctive transcriptional signature we found could potentially help predict severity of disease and guide treatment. Our findings also shed new light on mechanisms of immune dysregulation in sepsis.

## Background

The immune system plays a pivotal role in maintaining the balance between health and disease. Profiling immunological perturbation holds potential for elucidating the pathogenesis of a wide range of diseases. Currently available high-throughput profiling technologies and emerging systems immunology analysis approaches enable the study of clinical samples on a system-wide scale and provide unbiased tools for investigation of immune responses [1,2]. Whole blood transcriptome profiling has been employed to investigate a wide range of conditions [3-5]. Plasma, which is a valuable source of potential biomarkers, is an attractive alternative for profiling molecular changes associated with disease pathogenesis and progression on a systems scale. However, robust, cost-effective and reproducible technologies needed for measuring plasma protein abundance on a systems scale are still lacking. Most prevalent is mass spectrometry, however this lacks well-established reference databases and is biased toward detecting high-concentration compounds, which are major limitations for assessment of the plasma proteome by this technology [1,6].

So-called “transcriptomic reporter assays” provide an alternate means to assess perturbations in plasma on a system-wide scale [7]. This strategy consists of measuring whole genome transcriptional responses elicited in reporter cells exposed *in vitro* to patient plasma. This type of approach has already proven useful in studies of several immunologically mediated diseases. It was employed to help unravel the pathogenesis of systemic onset juvenile idiopathic arthritis, eventually leading to the adoption of a novel therapeutic modality for treatment of this disease [7,8]. It has been used to identify candidate biomarker signatures in patients prior to the clinical onset of type 1 diabetes mellitus [9]. It has contributed to identifying pathways of pancreatic islet cell destruction in islet cell transplantation [10,11]. Despite these and other successes, this approach has not yet been widely explored or adopted.

Sepsis is a clinical syndrome related to dysregulated systemic inflammation in response to an underlying infection. Uncontrolled production of cytokines and chemokines is believed to play a role in sepsis severity [12]. Early recognition leading to targeted antimicrobial and supportive therapy is critical to survival and each hour that treatment is delayed can markedly increase mortality [12-14]. However, due to an incomplete understanding of sepsis pathogenesis, criteria for rapid diagnosis and severity assessment are limited and based largely on non-specific clinical signs of systemic inflammation and organ dysfunction [15,16]. Several biomarkers have been studied in attempts to provide more simple, rapid, and accurate methods for diagnosis and prognosis of sepsis [17]. These include C-reactive protein, procalcitonin, triggering receptor expressed on myeloid cells 1 (TREM-1) and others [17-19]. While several small studies have shown correlation of such proteins to sepsis severity and outcomes, these proteins have not proven reliable on a larger scale and are not routinely used in clinical practice [18,20]. Recent studies suggest that combined use of multiple biomarkers may be more accurate, however at present these remain investigational [19].

Our study evaluated responses of three different cell types to stimulation with septic plasma: polymorphonuclear cells (PMNs), peripheral blood mononuclear cells (PBMCs), and monocyte-derived dendritic cells (MoDCs). PMNs and PBMCs were selected as these constitute the primary types of leukocytes in peripheral blood and are key in control of infections. MoDCs were selected because they are known to play a central role in the immune system and are able to respond to diverse immune signals. Each of these cell populations functioned as a so-called “reporter cell system” to investigate the transcriptional response to septic plasma. We demonstrate the utility of a reporter cell system for elucidating immune pathogenesis of a complex disease such as sepsis and the potential relevance of this approach for predicting prognosis in sepsis.

## Methods

### Ethics statement

The study was approved by the ethical review committees of Khon Kaen University and Khon Kaen Regional Hospital (Khon Kaen, Thailand) and the Institutional Review Board of Benaroya Research Institute (Seattle, WA). Participants provided written informed consent to participate in this study. Written informed consent was obtained from parents or guardians on behalf of the minor/child participant in this study.

### Plasma collection

Septic patients were enrolled from Khon Kaen Regional Hospital, Khon Kaen, Thailand. Patients who met at least two of the criteria for severe inflammatory response syndrome (SIRS) were enrolled in the study [3,15]. As part of the routine investigations, clinical specimens were collected for bacterial culture within 24 h following SIRS diagnosis. Only blood samples obtained from patients who were retrospectively diagnosed with culture-proven sepsis were retained for further analyses. Patients with negative blood cultures were excluded. Severe sepsis was defined according to the current guidelines from the Surviving Sepsis Campaign [15]. These criteria include several clinical and laboratory findings of sepsis-induced tissue hypoperfusion or organ dysfunction. We used the subset of these criteria for which the necessary data had been collected for our patient cohort: elevation in creatinine to >2.0 mg/dl, elevation in bilirubin to >2.0 mg/dl, platelet count <100,000/μL, and sepsis induced hypotension (septic shock). In hospital death was also counted as severe sepsis. Demographic and clinical data were recorded for all subjects (Additional files 1, 2, and 3). Uninfected healthy controls were selected as individuals who had no signs of acute infectious diseases during the previous 3 months or at the time of the study. Uninfected controls also had to have normal blood counts, normal fasting blood glucose, and normal glycosylated hemoglobin. Three milliliters of whole blood was collected from each patient and healthy control into heparinized tubes (BD Biosciences). For sepsis patients, samples were grouped as drawn either in the first 48 h of admission, or at >48 h after admission. To separate plasma, blood samples were centrifuged at 2,000 rpm for 10 minutes and the plasma component was transferred into a cryogenic vial and stored at -80°C until used.

### PMNs and PBMCs isolations from healthy volunteers

Blood samples from three additional healthy volunteers were used in subsequent cell isolation procedures. PMNs were isolated from heparinized venous blood by 3.0% dextran T-500 sedimentation and Ficoll-Paque PLUS centrifugation (Amersham Biosciences) as previously described [21]. The purity of isolated cells was generally more than 95% as determined by flow cytometry (FACSCalibur, Becton Dickinson) [22]. PBMCs were isolated from whole blood samples by centrifugation through a Ficoll-Paque Plus (Sigma Aldrich) density gradient.

### Generation of MoDCs

A portion of the isolated PBMCs was subsequently used for MoDCs generation as previously described [22,23]. MoDCs were harvested and resuspended in serum-free RPMI-1640 medium (Gibco), 5 × 10^5^ cells/well were plated into a 24-well tissue culture plate (Corning) for 24 h. The resulting cells were determined to be >95% CD11c^+^ by flow cytometry.

### Cell culture

Cell cultures were performed as described by Pascual *et al*. [7]. Two million PMNs or one million PBMCs were resuspended in serum-free RPMI-1640 medium (Gibco) and added to either 5 ml or 2 ml culture tubes (Becton Dickinson), respectively. Five hundred thousand MoDCs were seeded into 24 well tissue culture plates (Corning) at 1 × 10^6^ cells/ml and rested for 24 h before the experiments. Cells were cultured with medium alone or a plasma sample in a final concentration of 20%. After 6 h incubation at 37°C in 5% CO_2_, cells were harvested, washed twice with phosphate buffered saline, homogenized in RLT buffer (RNeasy mini kit; QIAGEN), and stored at -80°C until use.

### RNA preparation and microarray

Total RNA was isolated using the RNeasy Mini kit (QIAGEN) according to the manufacturer’s instructions. RNA integrity number (RIN) was determined by using an Agilent 2100 Bioanalyzer (Agilent). Qualified samples (RIN >6 or presence of 28s and 18s rRNA) were retained for further processing. Total RNA was amplified and labeled using the Illumina TotalPrep RNA Amplification Kit (Ambion). Labeled cRNA was hybridized overnight to Human HT-12 V4 BeadChip array (IIlumina), washed, blocked, stained and scanned on an Illumina HiScan instrument following the manufacturer’s protocols.

### Data acquisition and background subtraction

GenomeStudio was used to generate signal intensity values from the scans and perform background subtraction. Post-hybridization quality controls were done by the standard metrics provided by the manufacturer. Data from each cell type and each culture experiment were processed independently. All possible outliers were excluded from the expression data set by metrics for post-hybridization quality controls.

### Data normalization

All data analyses were performed using R (version 2.14.0; http://cran.r-project.org/bin/windows/base/old/2.14.0/). Data pre-processing of background subtracted data was performed by using the preprocessCore package from Bioconductor. Pre-processing included rescaling intensity by quantile normalization. After normalization, expressions were floored with intensities <10 set to 10. Transcripts with detection p-value of less than or equal to 0.01 in at least one sample (PALO) were selected for further analysis. Samples from the same cell type and batch were normalized to the average intensity of samples cultured in medium alone. A filter was set to include only transcripts that had at least two-fold changes and 100 intensity differences compared to medium control. Background subtracted and processed data from these experiments have been deposited at NCBI’s Gene Expression Omnibus database (http://www.ncbi.nlm.nih.gov/geo/), with accession numbers GSE49758. To facilitate data sharing and interactive data analysis, we created a data portal (https://gxb.benaroyaresearch.org/tra/tra-paper/tra-landing.gsp) to store and analyze background subtracted data from all three experiments (see [24] for tutorial).

### Unsupervised analysis

Principle component analysis (PCA) was performed using the R function “prcomp”. The first two principal components, PC1 and PC2, were plotted against each other. Each colored dot represents an individual sample. Euclidean distances were calculated by measuring the distance from each sample to the average of samples stimulated with uninfected plasma. The comparison between severe and non-severe sepsis was performed using the Mann-Whitney *U*-test. Hierarchical clustering analysis was performed using the function “heatmap.2” from the R package “gplots”. Euclidean distance and complete linkage methods were used by default.

### Feature selection

Transcripts that were differentially expressed between study groups were selected by Random k-Nearest Neighbor – Feature Selection (RKNN-FS) using the R package “rknn” [25]. A RKNN classifier consists of an ensemble of base k-nearest neighbor models (number of neighbors = 5), each constructed from a random subset of the input variables. Features were selected by ranking the importance of the PALO transcripts.

### Pathway analysis

Gene ontology analyses were performed using GeneGo MetaCore pathway analysis tool (Thomson Reuters, NY). The default background gene list was used for all enrichment analyses including process networks, pathway map folders, and pathway maps. Pathway maps are the collection of pathways grouped into folders according to main cell processes, protein functions, and diseases. Map Folders are the collection of pathways grouped into folders according to main biological processes. Statistical significance was ascertained by using a threshold of false discover rate (FDR) <0.05. The network builder tool using the shortest possible path with no more than 2 steps was used to represent functional interactions. Upstream transcription factors were identified for lists of over-expressed genes using the Transcription Factors tool.

### Class prediction

To determine the performance of our predictive signature, class prediction of binary variables was carried out by support vector machine (SVM; package “e1071”) and random forest (RF; package “randomForest”) algorithms. These machine learning methods are robust, well-accepted and commonly used methods for class prediction. Receiver operating characteristic (ROC) curves were constructed using the R package “ROCR”. Area under the curve (AUC) and confidence intervals were calculated using the R package “Hmisc”.

## Results

### Septic plasma elicits transcriptional responses that can be measured on a systems scale in a cell reporter assay

**Figure 1:**
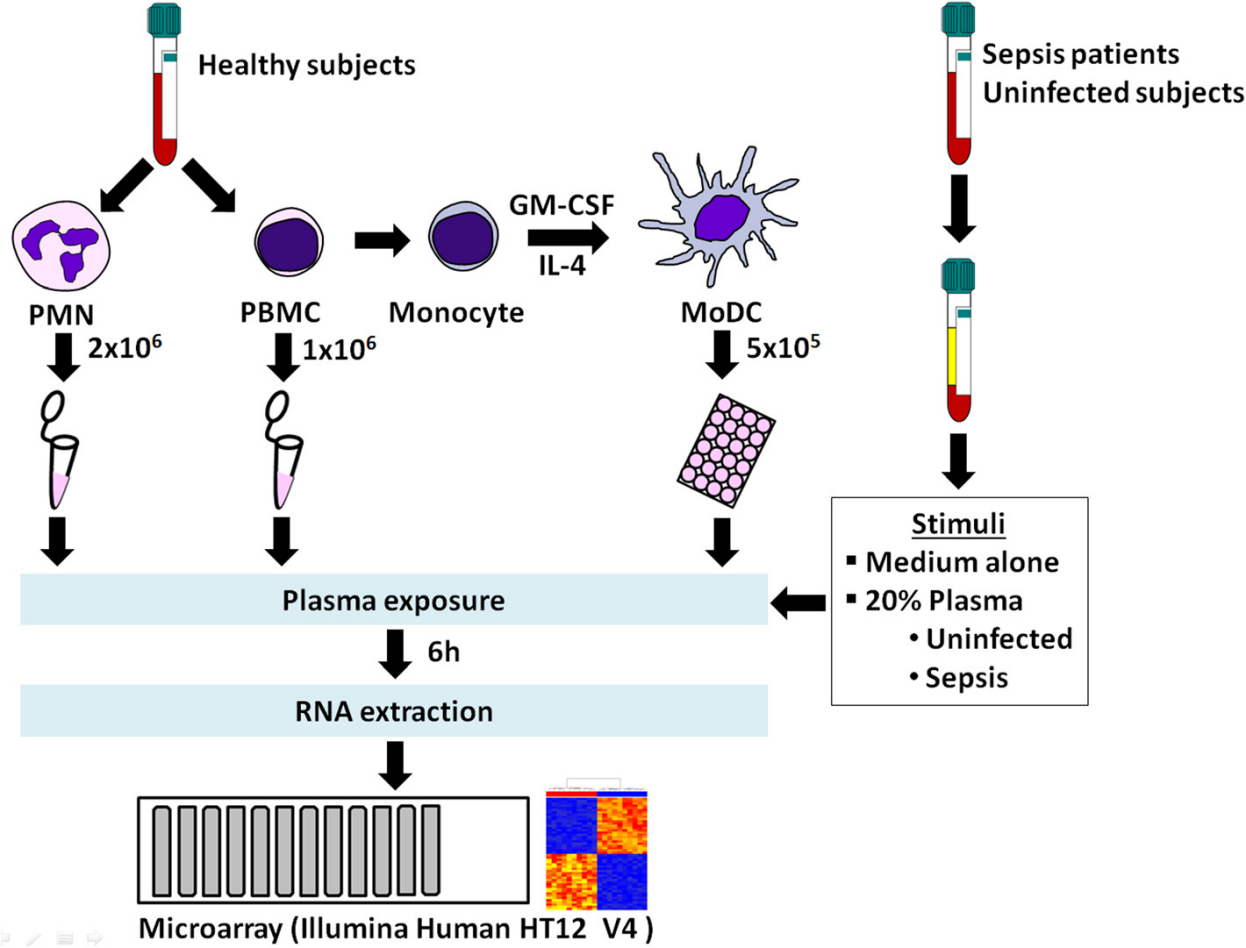
**Schematic illustration of the experimental design and procedures.** Experiments were performed using 3 independent sets of patient samples. Cells were isolated from healthy volunteers and the responses to plasma measured after 6 h of culture using whole genome Illumina Human HT-12 V4 BeadChips. Plasma samples were obtained from patients with culture-confirmed sepsis (n = 12, 29, and 35 in experiments I, II and III, respectively) and control subjects with no infection (n = 6, 20, and 20 in experiments I, II and III, respectively). In experiment I, three types of leukocyte populations were isolated from two healthy volunteers: PMNs, PBMCs and monocytes. In experiments II and III, only PMNs were used.

**Figure 2:**
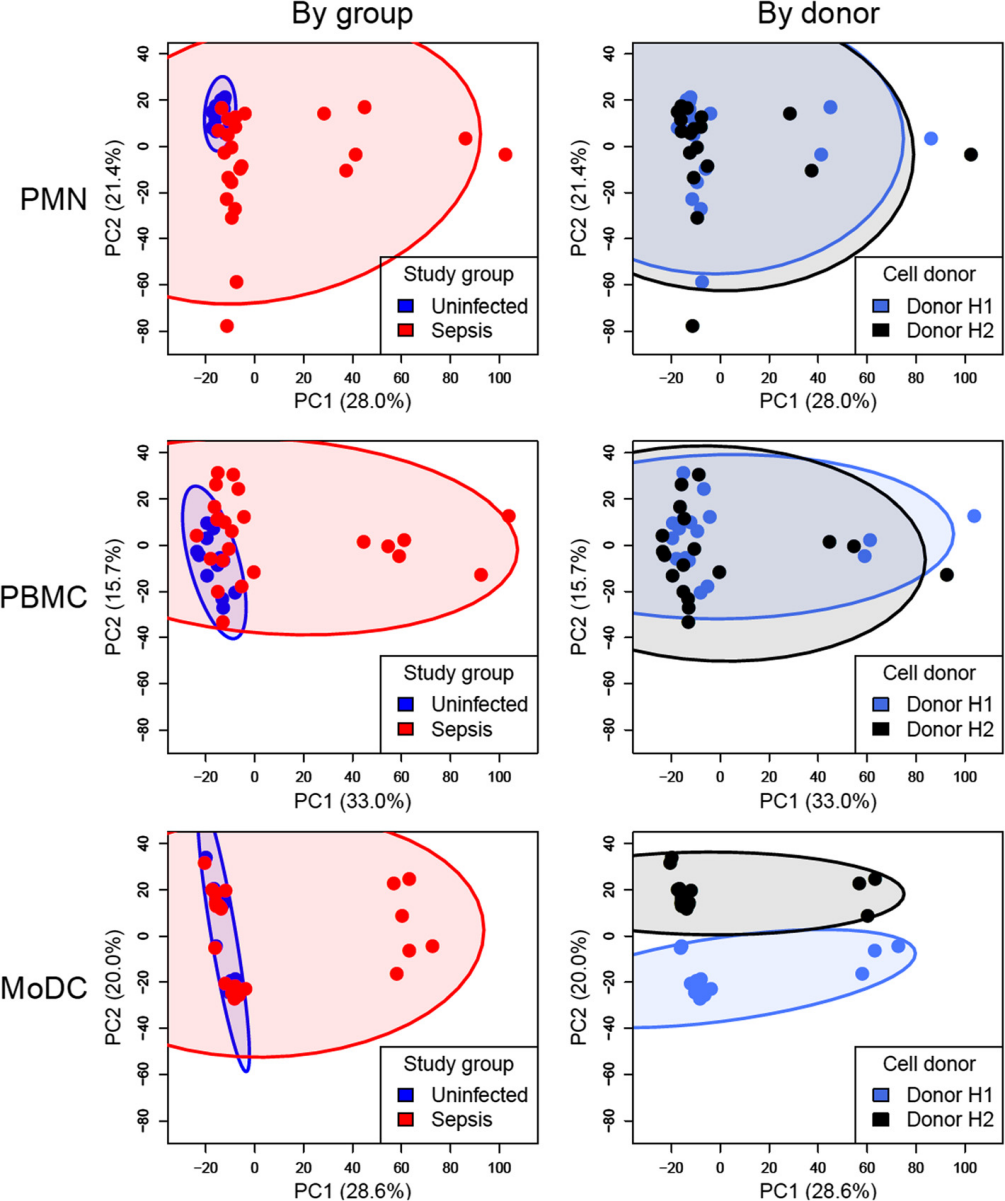
**Principal component analyses of transcriptional responses to septic plasma in three different reporter cells systems.** The transcriptional response to septic plasma measured by microarrays was analyzed separately for each reporter cell system. Fold-changes were calculated by normalizing the expression levels of cell cultures stimulated with plasma to their respective unstimulated cell cultures. Transcripts passing a filter criteria of 2 fold-change and 100 difference in intensity (2FC100DIF) were used for principal component analysis (PCA). Scores from principal component 1 (PC1) and principal component 2 (PC2), which explain approximately 50% of the variability, were plotted. Samples were color-coded according to study groups (blue = uninfected plasma; red = septic plasma; left panels) or cell donors (blue = donor H1; black = donor H2; right panels). Each ellipse indicates the 95% confidence interval of data from the corresponding group (indicated by colors). Numbers in parentheses indicate percentage of variance.

We first set out to determine which cell reporter system would be most amenable to detect meaningful changes in response to septic plasma *in vitro*. PMNs, PBMCs and monocytes were isolated from two healthy donors (Table 1). Monocytes were cultured with interleukin 4 (IL4) and granulocyte-macrophage colony-stimulating factor (GM-CSF) to generate MoDCs. Plasma samples were obtained from a first set of patients with culture-confirmed sepsis (n = 12) and from uninfected healthy controls (n = 6). Cells were cultured for 6 hours in medium alone (unstimulated) or in the presence of plasma (stimulated) using a final concentration of 20% (Figure 1). Microarray data were generated to assess transcriptional responses on a genome-wide scale. PALO filtering returned 21,236, 25,728, and 23,589 transcripts in PMNs, PBMCs, and MoDCs respectively. Transcripts were selected that changed by at least 2-fold and 100 intensity differences (2FC100DIF) in response to plasma stimulation compared to unstimulated cells. To reduce data dimensionality and facilitate the comparison among cell types, principal component analyses (PCA) were carried out. PCA plots of data obtained from the 3 different reporter cell systems (PMNs, PBMCs, and MoDCs) show changes in transcription that can be attributed to responses to plasma stimulation (Figure 2). The best separation between sepsis and uninfected plasma samples was observed in PMNs (Figure 2, top left panel). This result was confirmed by calculating the Euclidean distance from the center of uninfected controls (Additional file 4). These results demonstrate the feasibility of a “reverse proteomics” approach to detect the presence of immunomodulatory factors in the blood of sepsis patients. While responses measured in PMNs and PBMCs were consistent for both cell donors (Figure 2, upper and middle right panels), significant donor-to-donor variation was observed in MoDCs (Figure 2, lower right panel).

**Table 1:** Overview of experimental design and sample sizes

**Experiments**	**Cell donors**	**Plasma sources**
I	Healthy subjects (n = 2)	Uninfected subjects (n = 6)
		Sepsis subjects (n = 12)
II	Healthy subjects (n = 1)	Uninfected subjects (n = 18)
		Sepsis subjects (n = 29)
III	Healthy subjects (n = 2)	Uninfected subjects (n = 19)
		Sepsis subjects (n = 35)

### PMNs mount a robust immune transcriptional program in response to septic plasma

To test how our approach could best be used for monitoring immunomodulatory factors in the blood of sepsis patients, we next compared the transcriptional programs elicited by septic plasma in PMNs, PBMCs, and MoDCs. Septic plasma samples (n = 6/24) that were found to induce the most robust responses consistently across the three cell reporter systems were selected (Figure 2, Additional file 5). Transcripts changing by at least 2-fold compared to their own unstimulated controls in each reporter system (709, 366 and 452 transcripts for PMNs, PBMCs and MoDCs, respectively) were combined to generate a list of 1,366 transcripts (Additional files 6 and 7). The number of transcripts passing this 2-fold cut off was the highest in PMNs and the majority of these transcripts are over-expressed (Table 2). In contrast, there were fewer changes recorded in PBMCs and MoDCs and for the majority of those genes, transcript abundance decreased upon exposure to septic plasma.

**Table 2:** **Number of over**-**expressed and under**-**expressed transcripts induced by septic plasma**

**Cell types**	**Number of over**-**expressed transcripts**	**Number of under**-**expressed transcripts**
PMNs	465	358
PBMCs	168	442
DCs	125	488

In an effort to determine potential molecules present in septic plasma inducing these patterns of gene expression, we selected those genes over-expressed in each of the 3 reporter cell types and predicted the upstream transcription factors using GeneGo MetaCore. One hundred and sixty-one transcription factors were identified in total (Additional file 8). There was significant overlap among the 3 lists of transcription factors with 27 predicted for all 3 cell types, including several well established in core immune pathways such as *STAT3*, *STAT4*, *STAT5A*, *STAT5B*, and *CREB1*. These findings are consistent with the idea that a relatively small number of molecules are driving the observed transcriptional responses and could suggest future studies to determine the relevant immunomodulatory molecules in septic plasma.

**Figure 3:**
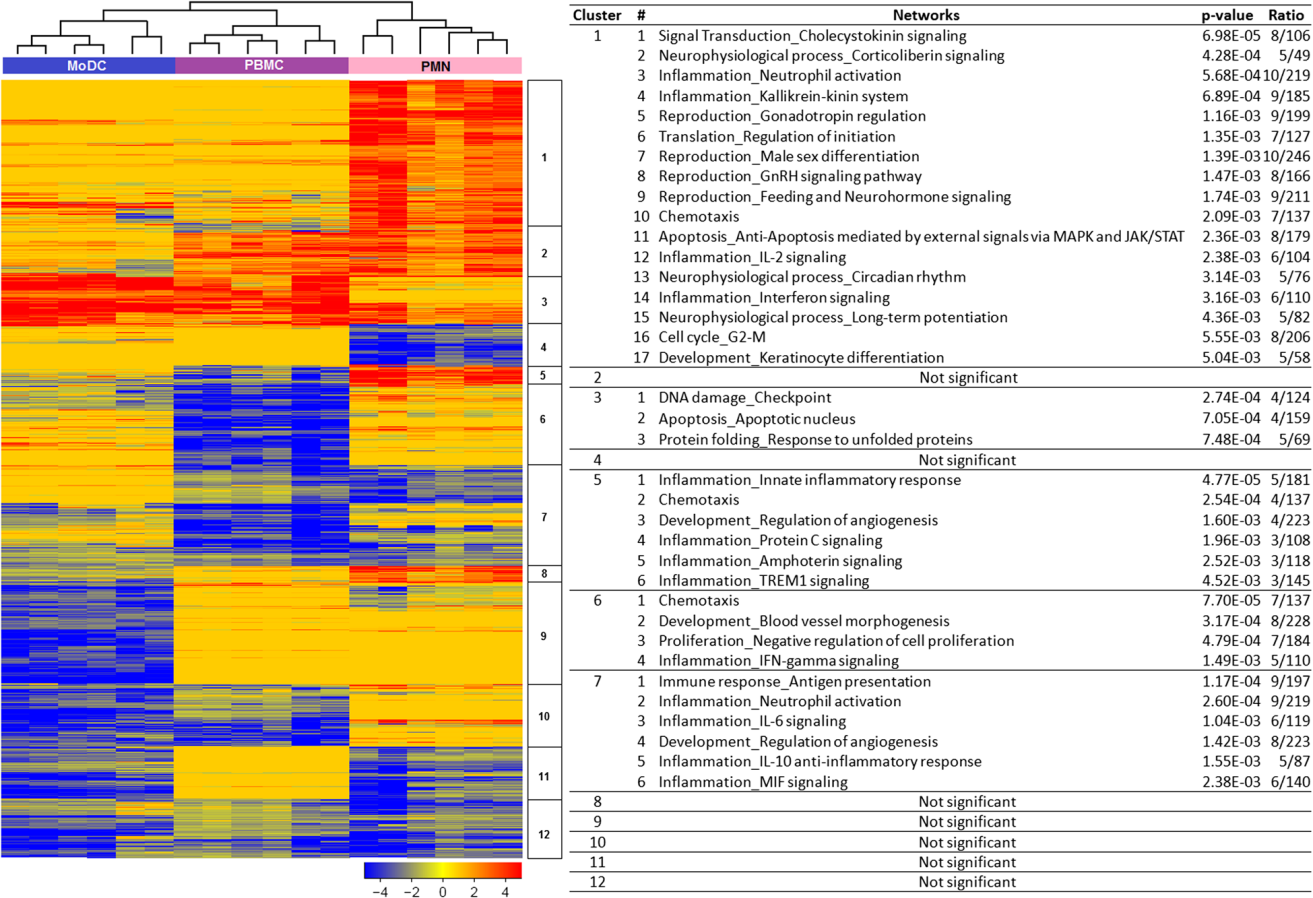
**Transcriptional programs elicited by septic plasma in three reporter cell systems.** Six samples, which provided the highest and most consistent response in all three reporter cell systems were selected (Additional file 5). Transcripts passing a filter criterion of 2 fold-change compared to medium controls were used (n = 1,366). Transcripts were organized by hierarchical clustering (Euclidean distance) according to similarities in expression profiles. Each row represents a transcript and each column an individual sample. The heatmap shows fold-change compared to unstimulated cell cultures. Red indicates over-expressed and blue indicates under-expressed transcripts. Clusters are identified by a number on the right side of the heatmap. Each cluster was annotated using the GeneGo MetaCore pathway analysis tool. P-values indicate enrichment significance for the networks based on hypergeometric distribution. Ratios indicate the number of molecules in the query set over the total number of molecules in the network.

To better understand the observed gene expression patterns and the functions of those genes, a heatmap was generated with the 1,366 transcripts differentially expressed in at least one cell type (Figure 3). We performed hierarchical clustering and further characterized the function of transcripts in each of the resulting clusters by using the GeneGo MetaCore pathway analysis tool. Networks with statistically significant enrichment of functional annotations were found in 5 out of 12 clusters. Most of the enriched functional networks are relevant to immune processes. Cluster 1 (n = 267), the largest cluster, is formed by transcripts over-expressed in PMNs. This cluster contains many genes involved in neutrophil function (e.g. *CD177*, *IL1R2*, *NLRP3*, *TNFSF8*, *FCGR1A*, *FCGR1B*, and *FCGR1C*). Cluster 3 (n = 84) contains transcripts over-expressed in PBMCs, MoDCs and to a lesser extent PMNs. This cluster contains genes enriched for cell cycle and apoptotic mechanisms (e.g. *CDKN1A*, *GADD45A*, *GAA45G*, *SMAD7*, etc). Cluster 5 (n = 39) is over-expressed in PMNs but under-expressed in PBMCs. This is a smaller cluster but it contains several important immune-related transcripts (e.g. *CCL2*, *CCL20*, *CXCL2*, *IL1RA*, *IL1A*, *IRAK3*, and *TLR2*). Innate immune pathways and chemotaxis are the major functions of the genes in this cluster; specific induction of these genes in PMNs is consistent with their known role as the immune cells that respond immediately to bacterial infection. Cluster 6 (n = 135) is under-expressed in PBMCs and is enriched in immune mediators involved in chemotaxis (e.g. *CCL3*, *CCL3L1*, *CXCL5*, and *CXCL16*), IFN-gamma signaling (e.g. *CASP5*, *FCAR* and *CFB*), and proliferation and morphogenesis (e.g. *MMP19* and *NRP1*). Lastly, cluster 7 (n = 178) is under-expressed in all reporter systems. The enriched functional networks for this cluster are relevant to both innate and adaptive immune responses, for example, antigen presentation (e.g. *CD58* and *STAT1*), and neutrophil activation (*IL6*, *NFKB2*, and *CXCL1*). Taken together, these results support the use of PMNs as a useful reporter cell type for sensing immunomodulatory constituents of septic plasma in our reverse proteomic assay system.

### A wide range of responses to septic plasma samples is observed in the PMN reporter assay system

**Figure 4:**
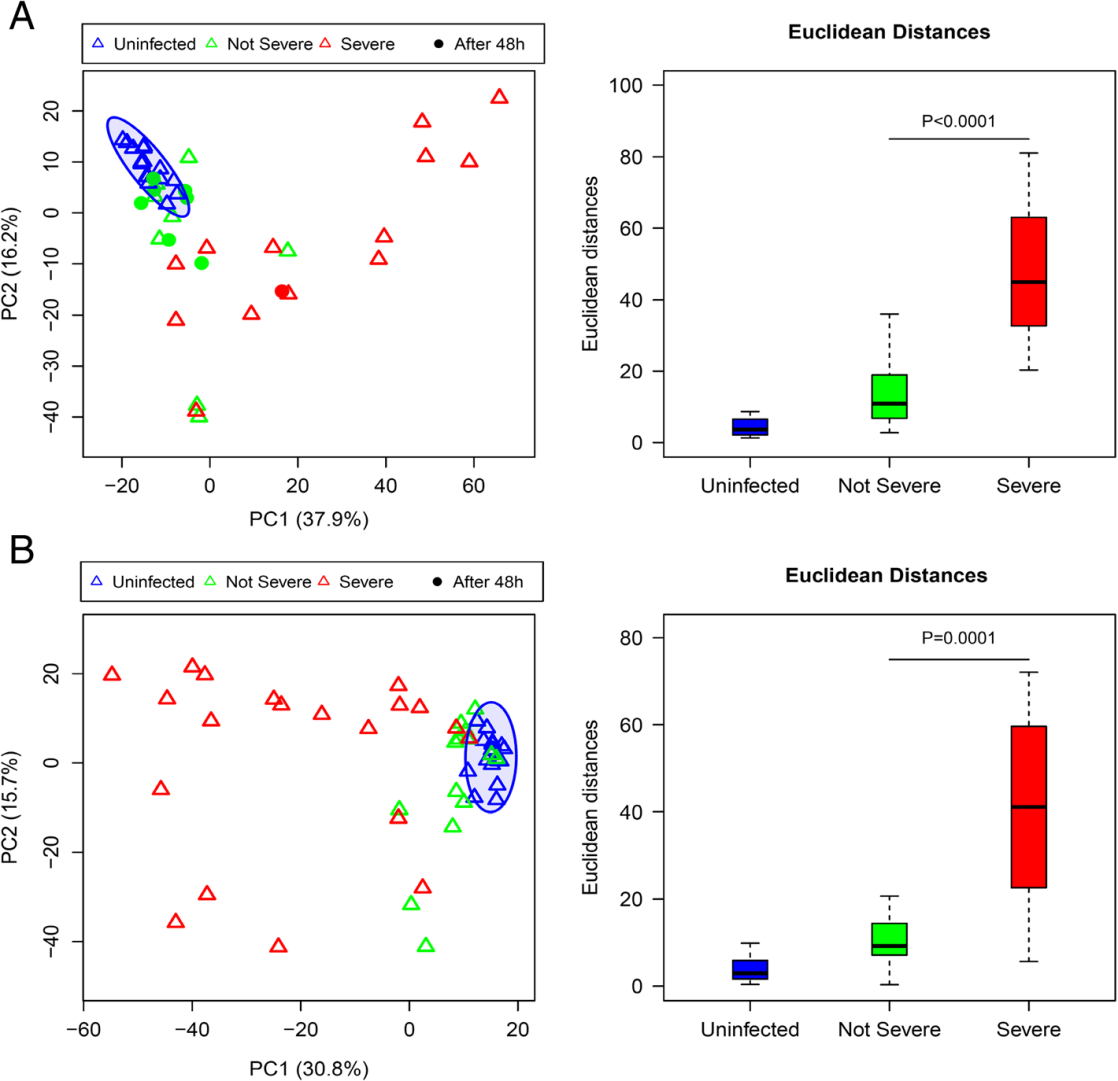
**Responses to septic plasma measured in a PMN reporter assay for additional sets of subjects.** Plasma samples from two additional sets of subjects were used in new sets of experiments: **A** and **B** corresponding to experiments II and III, respectively. PMNs used in both experiments were obtained from the same donor (donor H2) who had also participated in experiment I. Transcripts passing filter criteria of 2 fold-change and 100 difference in intensity (2FC100DIF) were used. PCA plots (left panels) were generated as described earlier. Colors and symbols indicate sample class and disease severity: red triangle, severe sepsis; green triangle, not severe sepsis; blue triangle, uninfected; and closed circle, plasma collected >48 h after admission. Numbers in parenthesis indicate percentage of variance. Box plots (right panels) show Euclidean distances calculated for each sample from the center of ellipses corresponding to responses to plasma from uninfected controls. P-values were derived from a Mann-Whitney *U*-test.

Next we sought to further investigate factors that account for the heterogeneity of the responses observed in PMNs exposed to plasma from the initial cohort of septic patients. Plasma samples from a larger independent set of septic (n = 29) and uninfected (n = 18) subjects were tested on PMNs collected from one of the cell donors used in our first experiment. PALO filtering returned 21,374 transcripts. The results were similar to that of the first experiment in both quantitative and qualitative aspects. The first two principal components, PC1 and PC2, explained more than 50% of the variability, and a distinct response to septic plasma compared with uninfected plasma was observed (Figure 4A, left panel). However, there was significant heterogeneity observed among the samples stimulated with septic plasma.

Further analyses identified 2 parameters accounting for much of the observed heterogeneity among samples stimulated with septic plasma – sepsis severity and timing of sample collection. To stratify severity, patient samples were categorized into “severe” or “not severe” sepsis according to predefined criteria (see Methods). In contrast to patients with non-severe sepsis, patients with severe sepsis were markedly separated from uninfected controls. This result was confirmed by calculating the Euclidean distance from the center of uninfected controls (ellipse in Figure 4A, right panel). The second important explanatory parameter identified was the timing of sample collection from hospital admission. There was no difference between transcriptional responses stimulated by septic plasma samples collected more than 48 h after admission and uninfected controls, whereas there was significant difference between septic plasma samples collected less than 48 h after admission and controls suggesting potential resolution of observed immunologic changes after treatment initiation. Numerous other demographic and clinical variables were investigated which did not appear to explain the observed heterogeneity (Additional file 9). The fact that most variability could be accounted for by sepsis severity suggests this is the primary factor determining the PMN response to plasma.

### Sepsis severity markers identified by the PMN reporter system

Since disease severity impacted the magnitude of transcriptional response to septic plasma, we evaluated the potential value of our PMN reporter system for clinical applications. We conducted a third experiment using PMNs from the same donor as in a second experiment with an independent set of septic plasma samples all collected within 48 h of admission (n = 35) and uninfected control samples (n = 19). PALO filtering returned 15,083 transcripts. The PCA plots generated from these transcripts showed similar results to that obtained in the second experiment confirming the marked separation of severe sepsis from not severe sepsis and controls and demonstrating sepsis severity the main variable affecting transcriptional responses (Figure 4B, Additional file 10). To assess variation of the cell donor source, we tested the same set of plasma samples with PMNs from an additional donor not used in either of the previous two experiments. PCA plots and Euclidean distance comparisons demonstrate similar findings as with the first donor suggesting this sort of PMN reporter system can be reproducible independent of the cell donor (Additional file 11).

**Figure 5:**
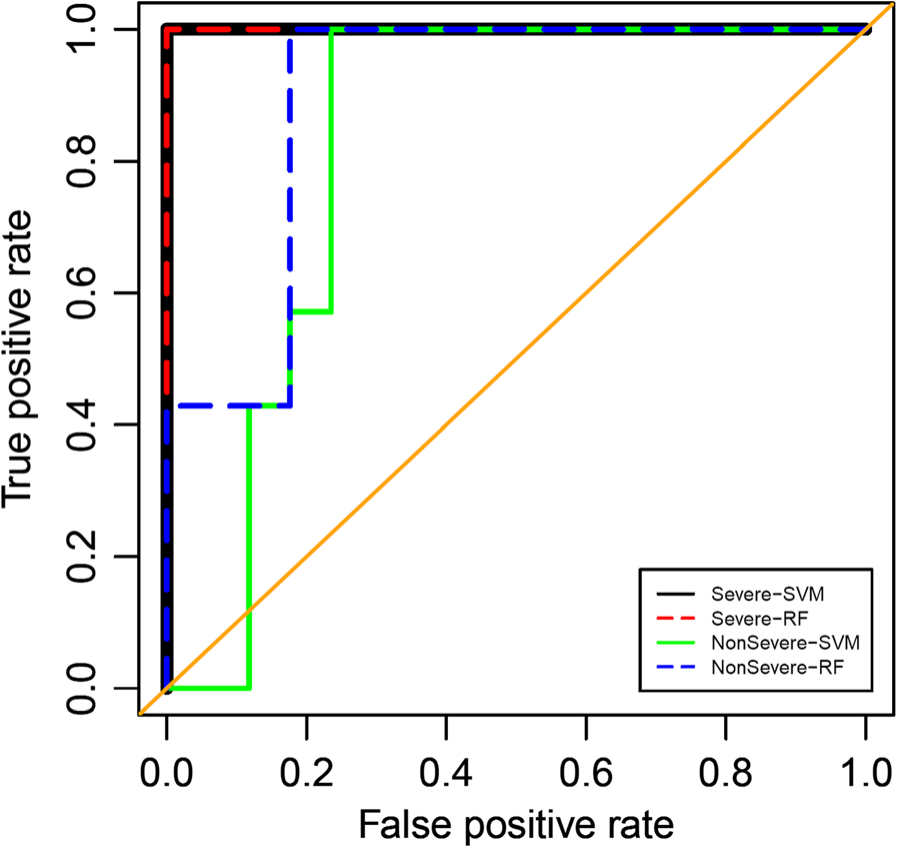
**Receiver operating characteristic (ROC) curve for the candidate septic severity biomarker signature.** This plot assesses the performance of the “septic severity” signature in predicting severe sepsis and non-severe sepsis. Areas under the curve (AUC) are 1 for the prediction of severe sepsis by both SVM and RF. AUCs are 0.82 (95% confidence interval, 0.78-1.00) and 0.90 (95% confidence interval, 0.66-0.99) for the prediction of non-severe sepsis by SVM and RF, respectively.

We then used a RKNN-FS algorithm to identify markers for differentiating severe and not severe sepsis (Table 3). Data from the third experiment were used as the training set because it was the most balanced and largest dataset (severe sepsis n = 20; not severe sepsis n = 15). RKNN-FS identified a set of 30 transcripts as being able to provide the highest accuracy (82.02%) in predicting sample class in a leave one out cross-validation scheme. This biomarker signature reflects different amplitudes of PMN responses to septic plasma samples according to disease severity. Misclassification was observed only with some non-severe sepsis and uninfected controls. This result is due to the more similar transcriptional response between these two groups as seen in Figure 4. To illustrate the performance of the identified markers for predicting both severe and non-severe sepsis from uninfected controls by a ROC curve, we built prediction models by using SVM and RF algorithms. Data from experiment II (severe sepsis n = 17; not severe sepsis n = 12) were used as a test set. The results demonstrated the high accuracy of the 30-transcript panel in predicting the response to plasma from severe sepsis samples; AUCs are 1 for both SVM and RF. The prediction accuracies of response to plasma from non-severe sepsis samples are slightly lower; the AUCs are 0.82 (95% confidence interval, 0.78 – 1.00) and 0.90 (95% confidence interval, 0.66 – 0.99) for SVM and RF, respectively (Figure 5). Meaningful validation could not be done using data from experiment I because almost all of these plasma samples (11/12) were obtained from patients with severe sepsis, 3 of which were collected at >48 h after admission.

**Table 3:** Sepsis severity markers identified by the PMN reporter system

**No**.	**Abbreviation**	**Accession number**	**Gene name**	**Illumina ID**
1	*ALG10B*	GenBank:NM_001013620.3	Asparagine-linked glycosylation 10	ILMN_1730304
2	*ARID5A*	GenBank:NM_212481.1	AT rich interactive domain 5A	ILMN_1689700
3	*CCL22*	GenBank:NM_002990.3	Chemokine (C-C motif) ligand 22	ILMN_2160476
4	*CCND3*	GenBank:NM_001760.2	Cyclin D3	ILMN_1668721
5	*DMXL2*	GenBank:NM_015263.2	Dmx-like 2	ILMN_1705663
6	*ECHDC3*	GenBank:NM_024693.2	Enoyl Coenzyme A hydratase domain containing 3	ILMN_2072178
7	*EXOC5*	GenBank:NM_006544.3	Exocyst complex component 5	ILMN_1788625
8	*FAM195A*	GenBank:NM_138418.2	Family with sequence similarity 195, member A	ILMN_1730523
9	*FCGR2B*	GenBank:XM_938851.1	Fc fragment of IgG, low affinity IIb, receptor	ILMN_1804174
10	*FKBP5*	GenBank:NM_004117.2	FK506 binding protein 5	ILMN_1778444
11	*IL18R1*	GenBank:NM_003855.2	Interleukin 18 receptor 1	ILMN_1781700
12	*IL1R2*	GenBank:NM_004633.3	Interleukin 1 receptor, type II	ILMN_1758371
13	*KLF9*	GenBank:NM_001206.2	Kruppel-like factor 9	ILMN_1778523
14	*MCOLN2*	GenBank:NM_153259.2	Mucolipin 2	ILMN_1660462
15	*METTL6*	GenBank:NM_152396.2	Methyltransferase like 6	ILMN_1661998
16	*MMP9*	GenBank:NM_004994.2	Matrix metallopeptidase 9	ILMN_1796316
17	*NCRNA00120*	GenBank:NR_002767.1	AKIRIN2 antisense RNA1 (non-protein coding)	ILMN_3239856
18	*P2RY2*	GenBank:NM_176071.1	Purinergic receptor P2Y	ILMN_1723535
19	*PCYOX1*	GenBank:NM_016297.2	Prenylcysteine oxidase 1	ILMN_2113535
20	*PGM1*	GenBank:NM_002633.2	Phosphoglucomutase 1	ILMN_1800659
21	*PIBF1*	GenBank:NM_006346.2	Progesterone immunomodulatory binding factor 1	ILMN_1758111
22	*SEC24A*	GenBank:NM_021982.1	SEC24 family, member A	ILMN_2126832
23	*SLC15A3*	GenBank:NM_016582.1	Solute carrier family 15, member 3	ILMN_2085862
24	*SLC25A3*	GenBank:NM_002635.2	Solute carrier family 25, member 3	ILMN_2332713
25	*SMAP2*	GenBank:NM_022733.1	Small ArfGAP2	ILMN_1781468
26	*TFRC*	GenBank:NM_003234.1	Transferrin receptor	ILMN_1674243
27	*TLR2*	GenBank:NM_003264.3	Toll-like receptor 2	ILMN_1772387
28	*TNFRSF9*	GenBank:NM_001561.4	Tumor necrosis factor receptor superfamily, member 9	ILMN_1813379
29	*TPST1*	GenBank:NM_003596.2	Tyrosylprotein sulfotransferase 1	ILMN_1651950
30	*YIPF5*	GenBank:NM_030799.6	Yip1 domain family, member 5	ILMN_1714756

### Functional annotation of the candidate gene signature is a further indication of its relevance as a severity biomarker

**Figure 6:**
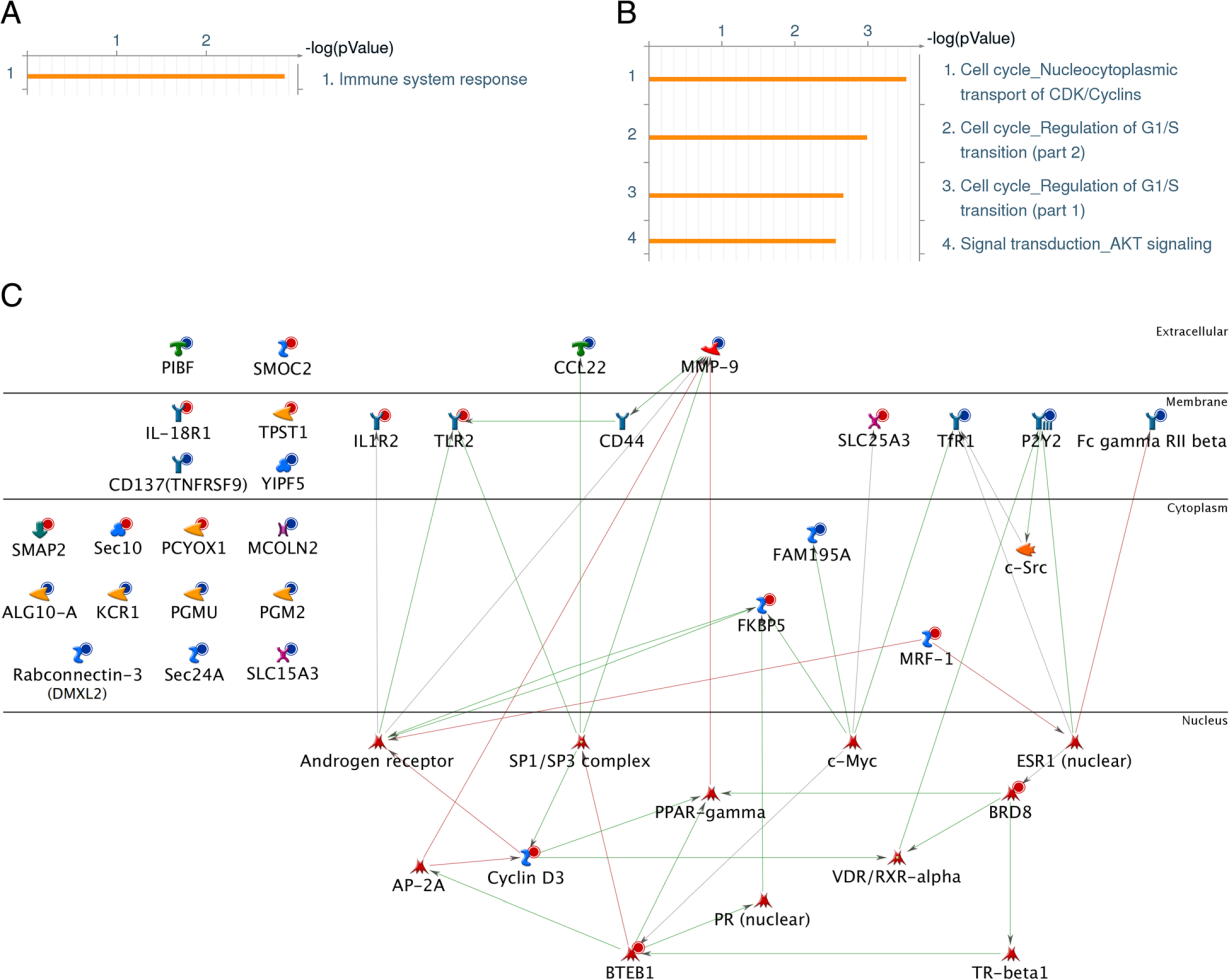
**Functional network for the septic severity biomarker signature.** Functional enrichment analyses were performed for the septic severity biomarker signature by using GeneGo MetaCore pathway analysis tool. Map Folders **(A)** and Pathway Maps **(B)** outputs are shown. Bars indicate the significance of the results (-log p-value). Only significant results were shown on the right of the bars. **(C)** A functional network was derived from the septic severity biomarker signature using GeneGo MetaCore. Compared to controls, input genes that are over-expressed and under-expressed in severe sepsis are indicated with red and blue circles, respectively. Red arrows indicate a negative interaction, green arrows indicate a positive interaction, and gray arrows indicate unspecified interactions.

Enrichment analyses were performed to characterize the functional relevance of our severity signature panel in GeneGo MetaCore. Collectively, these genes are involved in immune system response as their major biologic function (Figure 6A). Analyses for significant cellular process (Pathway Maps) also suggested their roles in cell cycle regulation (Figure 6B). The genes participating in these functions can be visualized on an interaction network (Figure 6C).

In addition to these enrichment analyses, many of our 30 classifier biomarkers have been previously shown to have potential roles in several dysregulated inflammatory conditions including sepsis and secondary organ dysfunction. Most of the 11 over-expressed transcripts have been independently identified as being of potential value as severity biomarkers in such inflammatory conditions. *FKBP5* has been shown to contribute to the regulation of myeloid-derived suppressor cells (MDSCs) and changes in MDSCs have been shown important in a sepsis model [26,27]. *TLR2* has been shown to be overexpressed in septic shock and also to play a role in acute kidney injury, which is an important consequence of severe sepsis and was part of our severity criteria [28,29]. *TPST1* over-expression can be induced by lipopolysaccharides (LPS) and inhibition of *TPST1* has been shown to affect macrophage signaling suggesting its role in the innate immune response [30]. *ECHDC3* over-expression was reported in onset of acute coronary syndrome [31]. *IL18R1* and *IL1R2* belong to the IL1R family. *IL18R1* plays a role in neutrophil migration and activation and has been identified as a biomarker for several systemic inflammatory conditions, such as surgery-induced inflammation and bacterial meningitis [32-34]. *IL1R2* expression has been suggested to be a marker of sepsis and high circulating IL1R2 protein levels have been reported in critically-ill patients with sepsis [35,36].

The functional relevance of the 19 under-expressed biomarkers was also investigated. *MMP9* has been shown to have negative correlation with multiple organ dysfunction scores in sepsis [37]. *FCGR2B* deficient mice have been shown to have increased bacterial clearance and survival in sepsis [38]. *TNFRSF9* is thought to be down-regulated by TNF-α, and to enhance anti-apoptosis and subsequently induce inflammatory responses [39,40]. *CCL22* is involved in regulating immune response by recruiting T-helper cells and regulatory T cells and a sepsis model demonstrated *CCL22* playing a role in enhancing neutrophil activation and chemotaxis [41-43]. *P2RY2* has been shown to play a role in neutrophil chemotaxis in a mouse model and is also involved in the recruitment of PMNs to the lung resulting in acute lung injury in sepsis [44,45].

Several other genes in our severity panel are involved in cell cycle and proliferation, for example, *KLF9*, *CCND3*, *YIPF5*, *ALG10B*, and *ARID5A*. Taken together this literature shows well-established pathogenic roles in sepsis or other inflammatory conditions for much of our candidate biomarker panel and constitutes an external validation of our reporter assay system.

## Discussion

This study reports the development and implementation of a “transcriptomic reporter assay” designed to investigate the immunogenicity of septic patient plasma and to our knowledge is the first published study employing immune cells in a whole genome transcriptomic reporter assay for an infectious process. One previous study investigated a focused transcriptional response of 1700 transcripts in cardiac myocytes cultured with septic serum [46]. Whole transcriptomic reporter assays have been employed successfully in previous studies of autoimmune diseases and have contributed to the understanding of disease pathogenesis in systemic onset juvenile idiopathic arthritis and type 1 diabetes mellitus [7-11]. Our work helps further extend this technique into the infectious disease field and also confirms the utility of comparing performance of multiple leukocyte subsets.

Transcriptomic reporter assays such as this one are based on the fact that plasma carries diverse circulating immune mediators. Stimulation of immune cells by plasma can demonstrate the biological processes triggered by the immune responses of the host [7,9]. In the context of infection, a transcriptomic reporter assay may be detecting in part the responses triggered by exogenous molecules including pathogen associated molecular patterns (PAMPs) such as LPS, lipoproteins, and peptidoglycans [47]. However such an assay also reflects the responses triggered by diverse endogenous signaling molecules including cytokines and damage associated molecular patterns (DAMPs) among others [48].

Our work identified that for investigation of sepsis, PMNs served as the best reporter cells in a side-by-side comparison with PBMCs and MoDCs. PMNs were better sensors of immunostimulatory factors present in plasma, displaying improved ability to discriminate septic from uninfected subjects, and PMNs mobilized the most robust immune transcriptional program. This finding was not initially expected given that PBMCs have been to date the preferred “serum sensing” cell reporter system [9], and given that dendritic cells are well known for their sentinel role in the immune system and have ability to respond to a wide range of immune triggers. However our findings are consistent with the role of PMNs, which serve as the first line of the cellular innate immune response and are a major source of acute phase immune mediators [49,50]. Further our results support previous work investigating the robust transcriptional response of PMN in sepsis. For example, Wong *et al*. demonstrated from leukocyte transcriptional profiling in pediatric septic shock that the number of differentially expressed genes in PMNs was greater than in monocytes or lymphocytes suggesting repression of adaptive immunity gene programs in early sepsis [51]. De Kleijn *et al*. demonstrated a robust set of functional gene networksdifferentially expressed by PMNs after both *in vivo* and *ex vivo* exposure to LPS that related to extended survival and the regulation of inflammatory responses [52]. It should be noted that the arrays of receptors expressed by different immune cell types vary widely, and that while our report indicate that neutrophils are especially well equipped to respond to plasma from septic patients it may not be the most appropriate cell type in other settings.

The PMN cell reporter system coupled with whole transcriptome readout allowed identification of a severity signature for sepsis that was highly accurate in two independent datasets. Current guidelines for diagnosing sepsis and grading severity are based on multiple clinical parameters, which can be inaccurate and do not predict prognosis well [15]. Although many potential biomarkers including cytokines, coagulation factors, and several others have been investigated for sepsis diagnosis and prognosis, none have proved reliable enough to enter routine clinical practice and so there is a need for better markers for risk stratification of septic patients to guide treatment and prognosis [17-19]. Elucidating the biologic mechanisms that differ among sepsis patients will help advance this field [12,20]. The promising performance of the PMN transcriptomic reporter assay presented here to stratify patients with sepsis by severity offers a novel and attractive platform for the development of biomarker signatures in sepsis. Similarly, the ability to select a gene panel that was specific to stimulation with plasma from severe sepsis patients shows the utility of this method to better understand the immunopathogenesis of sepsis.

Additional investigation is warranted by these results. The use of three independent cohorts of patients demonstrates reproducibility, but investigation with profiling of longitudinal samples will be necessary to further validate our severity assessment and to determine its potential value in monitoring disease progression and the response to treatment. Seeing consistent results using three separate PMN donors suggests our results can be reproducible independent of donor source, however further studies are necessary to determine how much variability donor source could introduce. Similarly extending our study to stable cell lines such as the neutrophil-like HL-60 line could be useful to develop a standardized assay, an approach that has been previously investigated in the context of type 1 diabetes research [53]. Perhaps most important, comparison of transcriptional profiles elicited from similar clinical conditions due to sterile inflammation (e.g. non-infectious SIRS or major surgery) is essential to determine the specificity of the severity signature described in this study. Also further work towards using this approach to identify a causative pathogen could hold potential – in our case the relatively small sample size and diversity of pathogens made it difficult to address this issue.

While at present a transcriptomic PMN reporter assay is not ideal for applications at the bedside given challenges for standardization of cell lines and data processing, technological advances in automation of sample processing and availability of polymerase chain reaction (PCR)-based amplification would make the implementation of a similar but more targeted assay feasible [54,55]. This approach could also serve as a novel platform for biomarker discovery and the development of novel clinical tests that could improve diagnosis and prognosis in sepsis. Moreover, this type of neutrophil transcriptomic reporter assay is likely to prove valuable for the investigation of other immunologically mediated diseases.

## Conclusions

We demonstrated the utility and accuracy of a neutrophil reporter assay coupled with whole transcriptome readout for predicting sepsis severity. We also demonstrated that this technique identifies important functional networks involved in the pathogenesis of sepsis.

## Abbreviations

AUC: Area under the curve; DAMP: Damage-associated molecular pattern; KNN: K-nearest neighbor; LPS: Lipopolysaccharides; MODC: Monocyte-derived dendritic cell; NCBI: National Center for Biotechnology Information; PALO: Presence in at least one sample; PAMP: Pathogen-associated molecular pattern; PBMC: Peripheral blood mononuclear cell; PC: Principal component; PCA: Principal component analysis; PCR: Polymerase chain reaction; PMN: Polymorphonuclear cell; RF: Random forest; RKNN-FS: Random k-nearest neighbor – feature selection; ROC: Receiver operating characteristic; SIRS: Systemic inflammatory response syndrome; SVM: Support vector machine.

## Competing interests

The authors declare that they have no competing interest.

## Authors’ contributions

DC and GL designed and conceived research; DR, DS, SB, CK, KO, and QAN performed experiments. WS provided specimens and clinical data. PK, EW, MCA, SP, and MM analyzed data. VG contributed new reagents/analytic tools. PK, DR, LC, MCA, PL, GL, and DC wrote the paper. All authors read and approved the final manuscript.

## Supplementary Material

Additional file 1: Table S1 Demographic and clinical data for subjects used in experiment I. Click here for file

Additional file 2: Table S2 Demographic and clinical data for subjects used in experiment II. Click here for file

Additional file 3: Table S3 Demographic and clinical data for subjects used in experiment III. Click here for file

Additional file 4: Figure S1 Box plot showing Euclidean distances from the PCA plots on Figure 2. Euclidian distances were calculated for each sample from the center of the ellipses corresponding to responses to plasma from uninfected controls in each reporter system (See Figure 2). Reporter cells and types of plasma are indicated on the x-axis. P-values were derived from a Mann-Whitney *U*-test. Click here for file

Additional file 5: Figure S2 Principal component analyses of transcriptional responses to septic plasma in three different reporter cells systems. A subset of septic plasma samples eliciting robust transcriptional responses consistently across all three cell reporter systems is indicated with red triangles on these PCA plots derived from Figure 2. Color indicates study groups (blue = uninfected plasma; red = septic plasma). An ellipsis indicates 95% confidence interval of data from the corresponding group (indicated by color). Number in parenthesis indicates percentage of variance. See the legend for Figure 2 for more details. Click here for file

Additional file 6 Expression profile of Transcripts changing by at least 2-fold compared to their own unstimulated controls in each reporter system. Click here for file

Additional file 7: Figure S3 Summary of transcripts expressed in each reporter cell system. Venn diagram demonstrating overlap of the 1,366 differentially genes (from Figure 3 and Additional file 6) for the 3 reporter cell types. Click here for file

Additional file 8 Predicted transcription factors from genes over-expressed in each reporter cell system. Click here for file

Additional file 9: Figure S4 Transcriptional responses and additional clinical data association (Experiment II). PCA plot from experiment II (Figure 4A) overlaid with additional clinical information for the sepsis patients: type of bacterial infection, Gram stain of bacterial infection, age (divided as <60 and ≥60 years-old), gender, presence of underlying diabetes mellitus, and presence of underlying chronic kidney disease. Click here for file

Additional file 10: Figure S5 Transcriptional responses and additional clinical data association (Experiment III). PCA plot from experiment III (Figure 4B) overlaid with additional clinical information for the sepsis patients: type of bacterial infection, Gram stain of bacterial infection, age (divided as <60 and ≥60 years-old), gender, presence of underlying diabetes mellitus, and presence of underlying chronic kidney disease. Click here for file

Additional file 11: Figure S6 Responses to septic plasma measured in a PMN reporter assay using PMNs from an additional healthy donor. Results from experiment III as shown in Figure 4B are replicated here in (A). PMNs from an additional donor were treated with the same set of plasma samples from experiment III (B) demonstrating similar responses. See Figure 4 legend for further details. Click here for file
