# Supplementary figures and images for: A transcriptomic reporter assay employing neutrophils to measure immunogenic activity of septic patients’ plasma
Source: J Transl Med. 2014 Mar 11;12:65. doi: 10.1186/1479-5876-12-65 (PMC4007645; doi:10.1186/1479-5876-12-65)

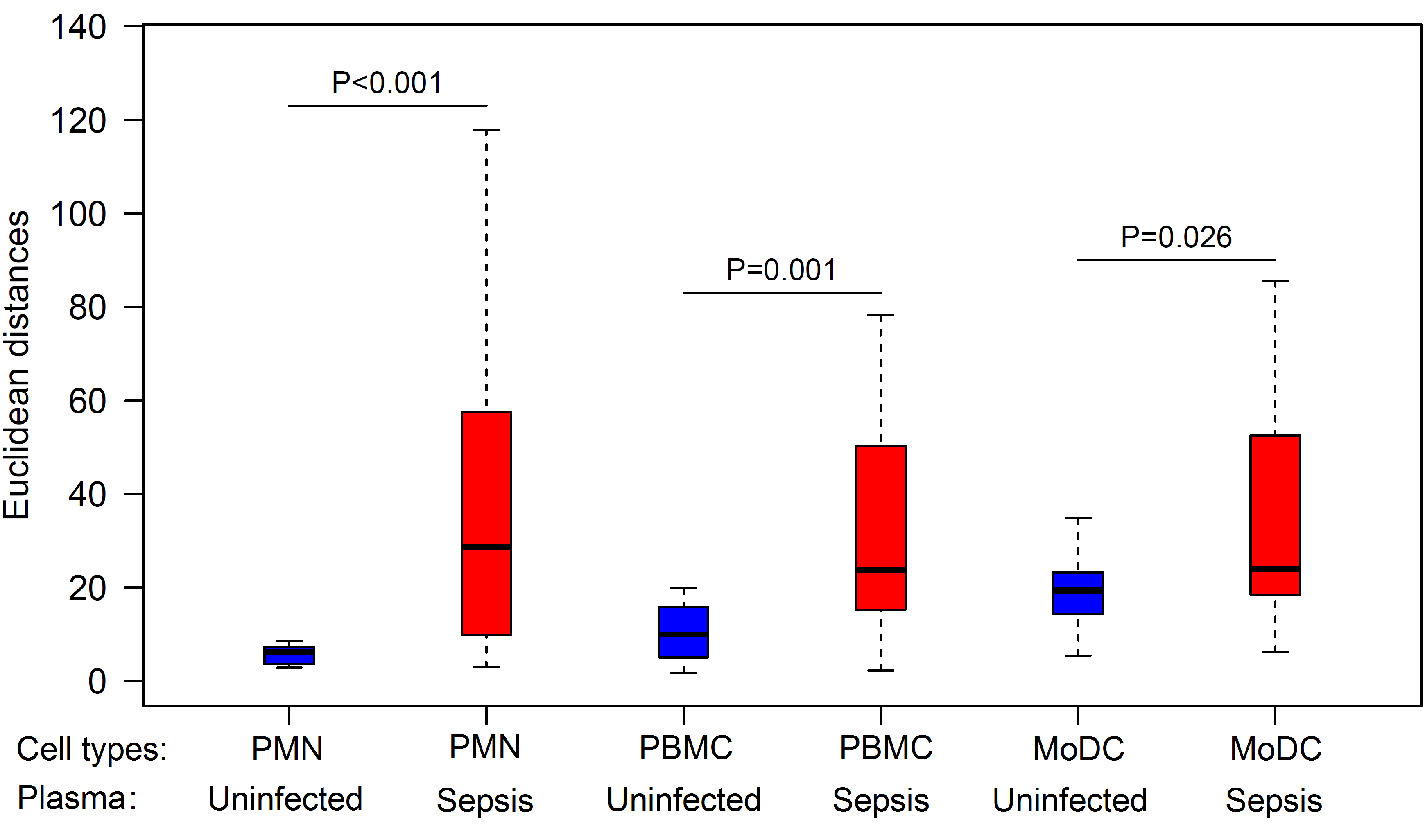

Supplement: Additional file 4: Figure S1 — Box plot showing Euclidean distances from the PCA plots on Figure 2. Euclidian distances were calculated for each sample from the center of the ellipses corresponding to responses to plasma from uninfected controls in each reporter system (See Figure 2). Reporter cells and types of plasma are indicated on the x-axis. P-values were derived from a Mann-Whitney U-test. [file 1479-5876-12-65-S4.tiff]

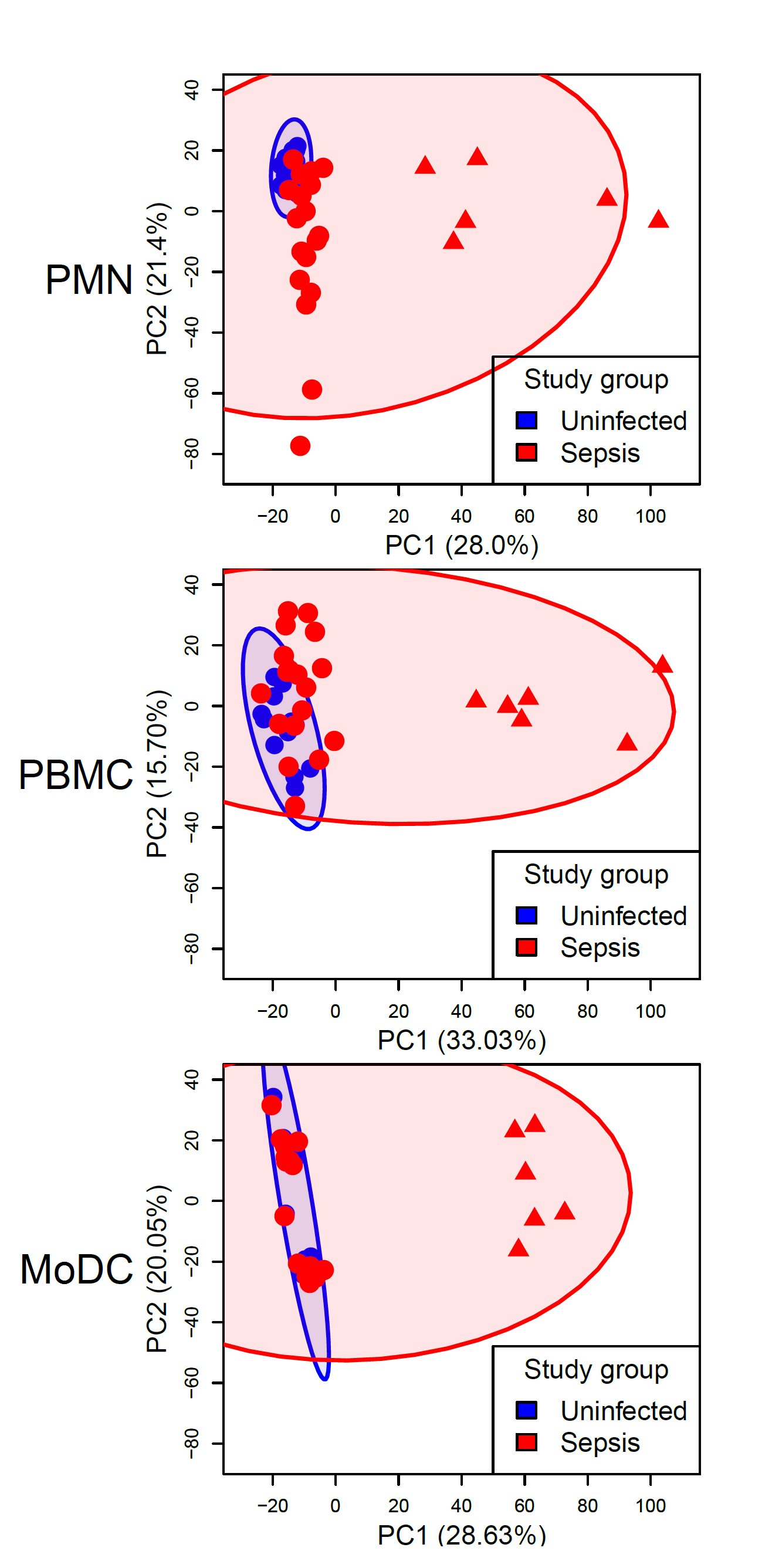

Supplement: Additional file 5: Figure S2 — Principal component analyses of transcriptional responses to septic plasma in three different reporter cells systems. A subset of septic plasma samples eliciting robust transcriptional responses consistently across all three cell reporter systems is indicated with red triangles on these PCA plots derived from Figure 2. Color indicates study groups (blue = uninfected plasma; red = septic plasma). An ellipsis indicates 95% confidence interval of data from the corresponding group (indicated by color). Number in parenthesis indicates percentage of variance. See the legend for Figure 2 for more details. [file 1479-5876-12-65-S5.tiff]

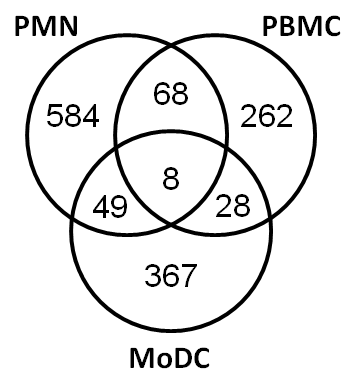

Supplement: Additional file 7: Figure S3 — Summary of transcripts expressed in each reporter cell system. Venn diagram demonstrating overlap of the 1,366 differentially genes (from Figure 3 and Additional file 6) for the 3 reporter cell types. [file 1479-5876-12-65-S7.tiff]

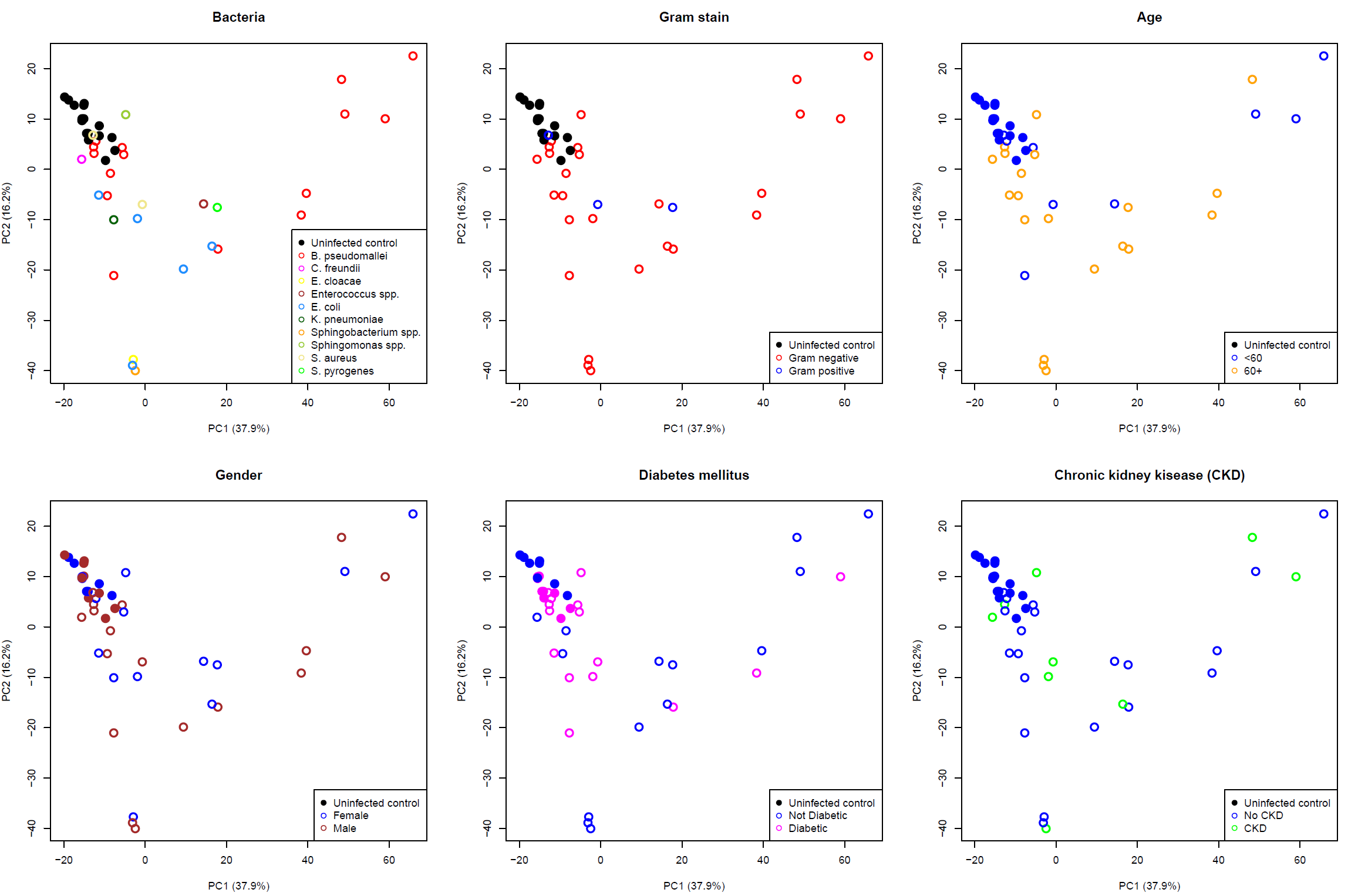

Supplement: Additional file 9: Figure S4 — Transcriptional responses and additional clinical data association (Experiment II). PCA plot from experiment II (Figure 4A) overlaid with additional clinical information for the sepsis patients: type of bacterial infection, Gram stain of bacterial infection, age (divided as <60 and ≥60 years-old), gender, presence of underlying diabetes mellitus, and presence of underlying chronic kidney disease. [file 1479-5876-12-65-S9.tiff]

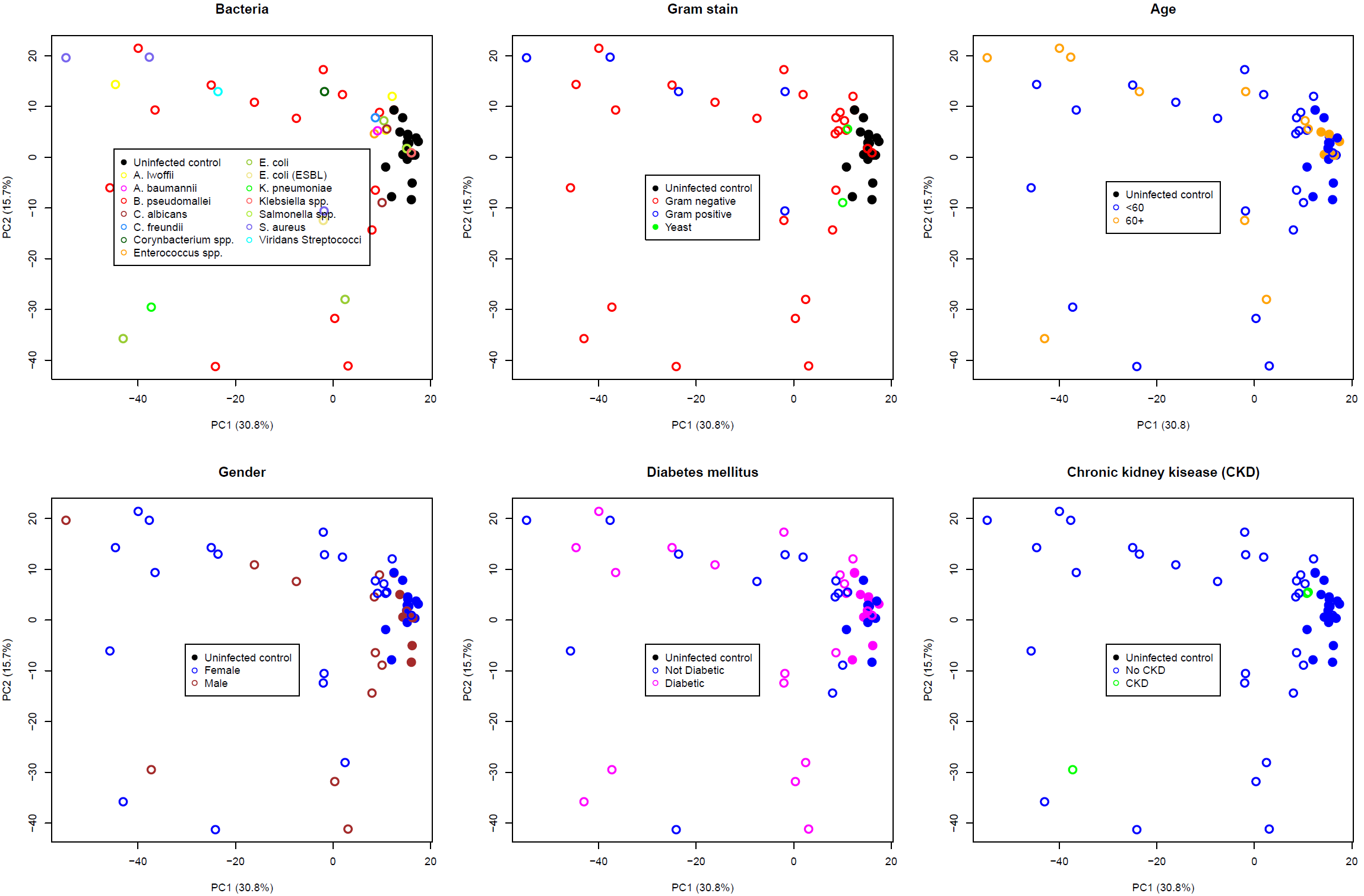

Supplement: Additional file 10: Figure S5 — Transcriptional responses and additional clinical data association (Experiment III). PCA plot from experiment III (Figure 4B) overlaid with additional clinical information for the sepsis patients: type of bacterial infection, Gram stain of bacterial infection, age (divided as <60 and ≥60 years-old), gender, presence of underlying diabetes mellitus, and presence of underlying chronic kidney disease. [file 1479-5876-12-65-S10.tiff]

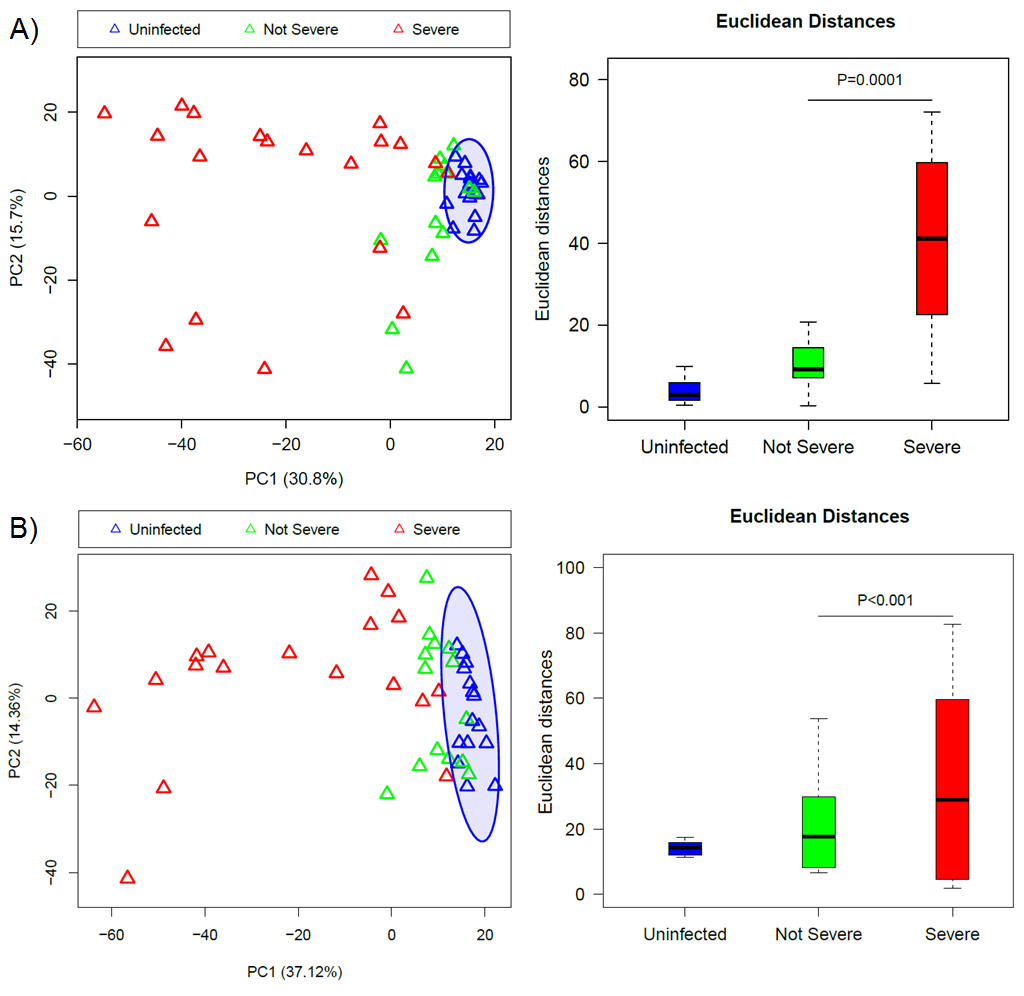

Supplement: Additional file 11: Figure S6 — Responses to septic plasma measured in a PMN reporter assay using PMNs from an additional healthy donor. Results from experiment III as shown in Figure 4B are replicated here in (A). PMNs from an additional donor were treated with the same set of plasma samples from experiment III (B) demonstrating similar responses. See Figure 4 legend for further details. [file 1479-5876-12-65-S11.tiff]
